# Effect of a probiotic beverage consumption (*Enterococcus faecium* CRL 183 and *Bifidobacterium longum* ATCC 15707) in rats with chemically induced colitis

**DOI:** 10.1371/journal.pone.0175935

**Published:** 2017-04-24

**Authors:** Larissa Sbaglia Celiberto, Raquel Bedani, Naiara Naiana Dejani, Alexandra Ivo de Medeiros, José Antonio Sampaio Zuanon, Luis Carlos Spolidorio, Maria Angela Tallarico Adorno, Maria Bernadete Amâncio Varesche, Fábio Carrilho Galvão, Sandro Roberto Valentini, Graciela Font de Valdez, Elizeu Antonio Rossi, Daniela Cardoso Umbelino Cavallini

**Affiliations:** 1 Universidade Estadual Paulista (UNESP), Faculdade de Ciências Farmacêuticas, Araraquara. Departamento de Alimentos e Nutrição, SP, Brasil; 2 Departamento de Tecnologia Bioquímico-Farmacêutica, Faculdade de Ciências Farmacêuticas, Universidade de São Paulo, São Paulo, SP, Brasil; 3 Universidade de São Paulo (USP), Faculdade de Medicina de Ribeirão Preto, Ribeirão Preto. Departamento de Bioquimica e Imunologia, SP, Brasil; 4 Universidade Estadual Paulista (UNESP), Faculdade de Ciências Farmacêuticas, Araraquara. Departamento de Ciências Biológicas, SP, Brasil; 5 Universidade Estadual Paulista (UNESP), Faculdade de Odontologia, Araraquara. Departametno de Fisiologia e Patologia, SP, Brasil; 6 Universidade de São Paulo (USP), Faculdade de Engenharia, São Carlos. Departamento de Hidraúlica e Saneamento, SP, Brasil; 7 Centro de Referencia para Lactobacilos, CERELA, S.M. Tucuman, Argentina; Wageningen University, NETHERLANDS

## Abstract

**Background:**

Some probiotic strains have the potential to assist in relieving the symptoms of inflammatory bowel disease. The impact of daily ingestion of a soy-based product fermented by *Enterococcus faecium* CRL 183 and *Lactobacillus helveticus* 416 with the addition of *Bifidobacterium longum* ATCC 15707 on chemically induced colitis has been investigated thereof within a period of 30 days.

**Methods:**

Colitis was induced by dextran sulfate sodium. The animals were randomly assigned into five groups: **Group C:** negative control; **Group CL:** positive control; **Group CLF:** DSS with the fermented product; **Group CLP:** DSS with the non-fermented product (placebo); **Group CLS:** DSS with sulfasalazine. The following parameters were monitored: disease activity index, fecal microbial analyses, gastrointestinal survival of probiotic microorganisms and short-chain fatty acids concentration in the feces. At the end of the protocol the animals’ colons were removed so as to conduct a macroscopical and histopathological analysis, cytokines and nitrite quantification.

**Results:**

Animals belonging to the CLF group showed fewer symptoms of colitis during the induction period and a lower degree of inflammation and ulceration in their colon compared to the CL, CLS and CLP groups (p<0.05). The colon of the animals in groups CL and CLS presented severe crypt damage, which was absent in CLF and CLP groups. A significant increase in the population of *Lactobacillus* spp. and *Bifidobacterium* spp. at the end of the protocol was verified only in the CLF animals (p<0.05). This group also showed an increase in short-chain fatty acids (propionate and acetate). Furthermore, the intestinal survival of *E*. *faecium* CRL 183 and *B*. *longum* ATCC 15707 in the CLF group has been confirmed by biochemical and molecular analyzes.

**Conclusions:**

The obtained results suggest that a regular intake of the probiotic product, and placebo to a lesser extent, can reduce the severity of DSS-induced colitis on rats.

## Introduction

Inflammatory bowel disease (IBD) comprise a group of complex disorders that affect the integrity of the intestinal mucosa. The most common forms of IBD are Crohn's disease (CD) and ulcerative colitis (UC). Its origin has not been fully elucidated, though it is believed that genetic susceptibility, intestinal dysbiosis and the immune system are involved in both its initiation and progression [1–3].

IBD pathogenesis involves the breakdown of intestinal mucosal homeostasis, which is associated with exacerbated inflammatory responses towards the intestinal microbiota [4,5]. Cytokines are the central components of IBD pathogenesis by mediating the crosstalk between immune and non-immune cells in the intestine. They are also involved in the recruitment of inflammatory cells to the intestinal mucosa [4–6].

Tumor necrosis factor alpha (TNF-α) is an immunoregulatory cytokine that favors inflammatory response by inducing the production of different mediators such as other cytokines, lipid metabolites and reactive oxygen species (ROS) [6]. The oxidative stress promoted by ROS [7] as well as the nitric oxide (NO) expressed by activated immune cells have both been associated with IBD development [6].

Interleukin-1 (IL-1) is another important cytokine produced by immune cells and is responsible for neutrophils recruitment, tumor development and the activation of innate lymphoid cells (ILCs). Moreover, IL-1 stimulates interleukin-6 (IL-6) production by macrophages, a pro-inflammatory cytokine that activates CD4^+^ T cells and macrophages, induces immune cells recruitment, generates epithelial cells proliferation and favors tumor growth [8].

The anti-inflammatory interleukin-10 (IL-10) may affect the intestinal inflammation since it is able to inhibit macrophages and T cell effector function. Increased levels of IL-10 have been observed in IBD patients [9]. In adittion, IL-10 knockout mice display spontaneous colitis as a result of the uncontrolled immune response stimulated by intestinal antigens [9,10].

Another anti-inflammatory cytokine, transforming growth factor-β (TGF-β), plays a critical role in a variety of cellular functions. For example, in the gastrointestinal tract, this cytokine has been associated with promoting a microenvironment which favors the suppression of inflammation and cancer development [11]. Although the treatment of patients with anti-inflammatory cytokines (IFN-β, IL-10 and IL-11) showed disappointing results [12–14], the use of infliximab, a TNF neutralizing antibody, in patients suffering from Crohn’s disease was rather promising [15]. To better understand the effect of probiotics in mediating inflammatory responses during colitis, pro-inflammatory and anti-inflammatory cytokines were investigated in this study.

Different strains of probiotic microorganisms have been investigated as an alternative for relieving the symptoms of IBD [16–19]. The mechanisms involved in the positive effects of probiotics in the context of IBD include pathogenic microorganism reduction, immune system modulation and the production of substances involved in cell proliferation and maturation, such as short-chain fatty acids (SCFA) [20–22]. In this line of thought, Medina et al. [23] showed that *B*. *longum* ATCC 15707 is able to beneficially modulate the immune system through IL-10 production and could therefore be used in controlling IBD.

Several studies concerning IBD and probiotics have demonstrated positive results using freeze-dried strains of bacteria, especially the genera *Lactobacillus* spp. and *Bifidobacterium* spp. [24–27]. However, these probiotic microorganisms can also be carried by the food matrix and therefore be incorporated as part of a varied diet. Thus, such probiotic foods could similarly contribute to reducing the risk of the IBD development in susceptible individuals.

Previous studies conducted by our research group have shown that a product developed from a soy aqueous extract and fermented with *E*. *faecium* CRL 183 and *L*. *helveticus* 416 has important functional properties that modulate immune responses, positively change the intestinal microbiota, and reduce the development of colon cancer in rats [28–30].

To the best knowledge of our research group, no studies are available in scientific literature regarding the impact of a soy-based fermented probiotic product on DSS-induced colitis. Therefore, the present study aims to investigate the effect of a soy-based product fermented by *E*. *faecium* CRL 183 (probiotic strain) and *L*. *helveticus* 416 with *B*. *longum* ATCC 15707 addition in rats with chemically induced colitis.

## Material and methods

### Production of the probiotic and placebo products

The soy-based fermented products were processed at UNIVERSOJA according to Rossi et al. [31]. The bacterial inoculum consisted of a equal mixture (1:1) of *E*. *faecium* CRL 183 (probiotic strain from Centro de Referencia para Lactobacilos–CERELA, S. M. Tucumán, Argentina) and *L*. *helveticus* 416 (fermentation adjuvant from Institute of Food Technology—ITAL—Campinas, SP, Brazil). Fermentation was carried out at 37°C until the product reached pH 4.5. After fermentation, a specific quantity of the strain of *B*. *longum* ATCC 15707 was added so as to yield 10^8^ CFU/g of product. These cells were inoculated in a milk medium (10% skimmed milk powder, 1% glucose, 0.5% yeast extract), and incubated overnight at 37°C, before being added to the product. The unfermented soy product (placebo) was chemically acidified with food-grade lactic acid (Purac, Sao Paulo, Brazil) in order to reach the equivalent pH of the fermented product. The products were freshly produced weekly and stored at 5°C.

Microbiological analyses of the fermented soy-based product revealed that the average population of *E*. *faecium* CRL 183, *L*. *helveticus* 416 and *B*. *longum* was 8 log CFU/g for all of the microorganisms throughout the experimental period.

### Experimental protocol

Male wistar rats (Specific Pathogen Free) weighing an average of 200 g were purchased from the central animal facility (CEMIB) at the State University of Campinas (Campinas, SP, Brazil). The rats were group-housed in a ventilated rack (Alesco, Brazil) that delivered HEPA filtered air to all cages, with controlled humidity (60%) and temperature conditions (20°C±2°C). The rats were exposed to 12-12h light-dark cycles, and were fed with a standard diet including drinking water *ad libitum*. The protocol performed followed the guidelines written by the Brazilian College of Animal Experimentation (COBEA) and it was approved by the Research Ethics Committee of the UNESP School of Pharmaceutical Sciences in Araraquara, Brazil (protocol number: 16/2012).

The animals were randomly assigned in 5 groups (n = 10):

**Group C:** healthy animals that did not receive the products under study;

**Group CL:** animals with chemically induced colitis that did not receive the products under study;

**Group CLF:** animals with chemically induced colitis that received the fermented product;

**Group CLP:** animals with chemically induced colitis that received the non-fermented product (placebo);

**Group CLS:** animals with chemically induced colitis that received sulfasalazine (drug widely used for UC treatment).

#### Dextran sulfate sodium induced colitis

Colitis was chemically induced by dissolving 4% DSS (MP Biomedicals, EUA; PM = 36.000–50.000) in the animals’ drinking water which has been ingested on a daily basis for a period of seven days. Individual body weight, total food and water intake, diarrhea and rectal bleeding were recorded daily for the duration of the study period. During the DSS administration, clinical signs were investigated such as pronounced weight loss (>20%), severe diarrhea, dehydration, general activity and any other symptons indicating the animal was at or near endpoint.

DSS-colitis severity was also determined daily using the disease activity index (DAI). This follows the methodology proposed by Murthy et al. [32], who takes into account three parameters: weight loss, stool consistency and occult bleeding in the feces. For each parameter, a score of 0 to 4 was attributed, thus resulting in the total DAI score. In order to determine the presence of fecal occult blood, the commercial kit Hemoplus (Newprov, Brazil) was used.

#### Administration of products

**Fig 1:**
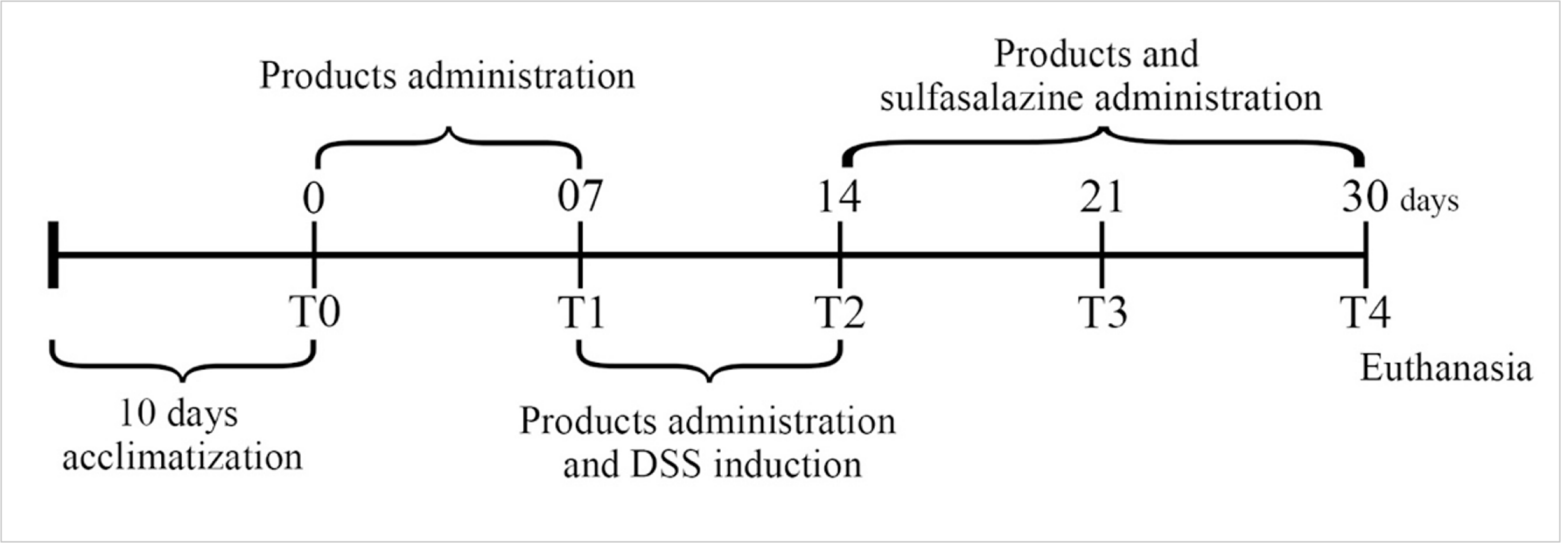
Colitis induction and products administration during the experimental protocol.

2 mL of the tested products were administered to the animals in groups CLF and CLP daily for 30 days by oral gavage. The administration of products had started out seven days before colitis induction and lasted for 16 days after the DSS induction (7 days), amounting to 30 days of treatment. CLS group received sulfasalazine (Sigma, USA) daily by oral gavage (100 mg / kg / weight, dissolved in 2 mL of sterile water) after colitis induction from the 14^th^ day (Fig 1). Animals belonging to the control (C) and colitis (CL) groups received 2 mL of sterile water by oral gavage daily.

#### Colon evaluation

After 30 days of experimentation, the animals were euthanized in a CO_2_ chamber and all colons were removed for further macroscopic and histopathological analysis. Samples were measured and weighted for inflammation assessment.

### Macroscopic and histopathological evaluation of DSS-colitis

Colon sections of the different experimental groups were photographed for a macroscopic analysis. According to Bell et al. [33] and Camuesco et al. [34], this was completed by considering the extent and severity of tissue damage. Each sample was awarded a score from 0 to 10 according to visible macroscopic damage.

Colon tissues were fixed in 10% buffered formalin, washed in tap water for 24 h and stored in 70°GL ethanol. Subsequently, the samples were routinely processed and embedded in paraffin. Distal colon tissues of approximately 5 μm were stained with hematoxylin—eosin and analyzed by light microscope (Olympus BX51—Olympus Optical, Tokyo, Japan) [17].

### Fecal microbial analyses

Different genera of the fecal microbiota were analyzed at the beginning of the experiment (T0 –before product administration), after 7 days of product ingestion (T1), after 7 days of colitis induction (T2), 14 days after colitis induction (T3) and at the end (day 30) of the experiment (T4). Stool samples were collected from the animals’ cages, corresponding to a 24 h period. The samples were, stored in sterile polypropylene bags and frozen at -80°C until the analysis.

The fecal microbial analyses were performed by media-dependent assay and based on the determination of the following bacterial populations: *Enterococcus* spp., *Lactobacillus* spp., *Clostridium* spp., *Bacteroides* spp., *Bifidobacterium* spp., and enterobacteria.

Fecal pellets were diluted in sterile peptone water and the suspensions were used to inoculate selective culture media: *Enterococcus* spp.: KF *Streptococcus* agar (Acumedia, USA), 37°C, 48 h [35]; *Lactobacillus* spp.: Man Rogosa Sharpe agar (MRS) (Acumedia), 37°C, 48 h [36]; enterobacteria: MacConkey agar (Acumedia), 37°C, 48 h [37]; *Clostridium* spp.: Reinforced *Clostridium* Agar (Acumedia), 37°C, 48 h [38]; *Bacteroides* spp.: *Bacteroides* bile esculin agar (BBE) (Acumedia), 37°C, 72 h [39]; *Bifidobacterium* spp.: *Bifidobacterium* iodoacetate medium 25 (BIM-25), 37°C, 72 h [40].

### ELISA

The levels of TNF-α, IL-10, IL-1β, IL-6 and TGF-β in colon homogenate supernatants were measured by using ELISA kits (BD Biosciences, San Jose, CA, USA; R&D Systems Inc, Minneapolis, MN, USA). All procedures were performed following the manufacturer’s instructions.

### Quantification of nitrite

Nitrite concentrations, an indicator of NO synthesis, were measured in the colon supernatant samples by using Griess reaction method, as previously described [41]. Samples absorbance was measured in a microplate reader with a 540 nm filter (Biotek, model 960).

### Determination of the short chain fatty acids

The fecal SCFA were extracted with diethyl ether [42] and their concentration was determined by gas chromatography (GC) according to Adorno et al. [43]. The results were expressed as total acetate, total propionate and total butyrate.

The samples were injected into a capillary column (HP-INNOWAX– 30m, 0.25nm, 0.25μm—Agilent Technologies) in a Shimadzu GC-2010 (Kyoto, Japan) gas chromatograph equipped with FID, a COMBI-PAL auto sampler using 10 mL headspace vials and a 2.5 mL HD type Hamilton gas-tight syringe.

The chromatographic conditions for the headspace method were similar to those proposed by Adorno et al. [43]: 35°C (0 min), 2°C/min at 38°C (0 min), 10°C/min at 75°C (0 min), 35°C/min at 120°C (1 min), 10°C/min at 170°C (2 min), with total run time of 14.49 minutes (min). The column flow was 1.5 mL ·min−1 with ultra-pure hydrogen (H_2_) as the carrier gas. The detector used was H_2_ at 30 mL·min−1 with synthetic air at 300 mL·min−1 and nitrogen (N_2_) at 30 mL·min−1 flows as flame and make-up gases, respectively. The temperatures of the detector and injector were held at 280°C and 250°C, respectively.

### Gastrointestinal resistance of *E*. *faecium* and *B*. *longum*

Stool samples were homogenized in sterile peptone water and serially diluted. Isolation of *Enterococcus* spp. and *Bifidobacterium* spp. colonies was performed by using the KF *Streptococcus* agar (Acumedia) and BIM-25 agar plus L-cisteyne 0.5% (InLab, Sao Paulo, Brazil) and the plates were incubated at 37°C for 48 h.

Isolated colonies of *Enterococcus* spp. and *Bifidobacterium* spp. with different morphologies were selected and transferred to the Bile Esculin Azide agar (Acumedia) and BIM-25 agar with the addition of L-cisteyne 0.5% (InLab), respectively. API 20 Strep and API 50 CHL kits (Biomerieux, France) were used to identify the species of *Enterococcus* spp. and *Bifidobacterium* spp., respectively. Colonies with a positive identification of *E*. *faecium* and *B*. *longum* were confirmed by PCR. The genomic DNA extraction was performed using the DNeasy Blood & Tissue Kit (Qiagen, USA).

#### PCR *Enterococcus faecium*

***Primers*:** Ent 1 (5’-ATTACGGAGACTACACACTTTG-3’) and Ent 2 (5’- TAGCCATAGAAGTTACATCAAG-3’) - 16S-23S rRNA [44]. **Reaction conditions:** 1x Taq DNA buffer, 1.5 mM of MgCL_2,_ 0.2 mM of each deoxynucleotides, 1 μM of each *primer*, 2 ng of genomic DNA and 2U of Taq DNA polymerase enzyme, in a final volume of 25 μL. The reactions were amplified using the following steps: 1 cycle at 94°C for 5 min, 30 cycles at 94°C for 1 min, 56°C for 30 s, 72°C for 1 min and a final extension at 72°C for 5 min [45,46].

#### PCR *Bifidobacterium longum*

***Primers*:** BiLON-1 (5’–TTCCAGTTGATCGCATGGTC–3’) and BiLON-2 (5’-GGGAAGCCGTATCTCTACGA-3’) - 16S rRNA. **Reaction conditions:** 1x Taq DNA buffer, 1.5 mM of MgCL_2,_ 0.2 mM of each deoxynucleotides, 1 μM of each *primer*, 2 ng of genomic DNA and 2U of Taq DNA polymerase enzyme, in a final volume of 25 μzL. The reactions were amplified by using the following steps: 1 cycle at 94°C for 5 min, 35 cycles at 94°C for 20 s, 55°C for 20 s, 72°C for 30 s and then 5 min at 72°C [47].

PCR products were visualized in an agarose gel electrophoresis (1% in TAE buffer– 40 nmol/L Tris, 11% glacial acetic acid, 1 mmol/L EDTA) using a 1 kb Ladder Plus (Invitrogen, USA) as molecular size marker. The gels were stained with SYBR Safe (Invitrogen, USA) and the images acquired under UV [45,46].

### Statistical analysis

The data were expressed as the mean value ± SD for each group. A statistical analysis was performed using the GraphPad Prism Software Version 6.0 (GraphPad Software, San Diego, California). The one-way analysis of variance (ANOVA) and Tukey’s multiple-comparison test were used to analyze the results. Significance was declared when p<0.05.

## Results and discussion

The probiotic soy product administered to the CLF group was fermented with mixed inoculum of *L*. *helveticus* CRL 416 and *E*. *faecium* CRL 183; however, only the *E*. *faecium* CRL 183 had its functional properties proven through *in vivo* and *in vitro* studies [28,29,44,48–50]. *L*. *helveticus* 416 was selected for technological reasons since preliminary testing conducted by our research group demonstrated a faster and more effective fermentation process when these two microorganisms are associated. The strain of *B*. *longum* ATCC 15707 was added to the product due to its immunomodulatory properties, as previously reported in literature [23].

During the 30 days of protocol, the fermented probiotic product showed more than 9 logCFU/g of the three organisms analyzed, which is considered appropriate for them to exert their probiotic effects [51,52].

### DSS-induced colitis symptoms are reduced in CLF group

**Fig 2:**
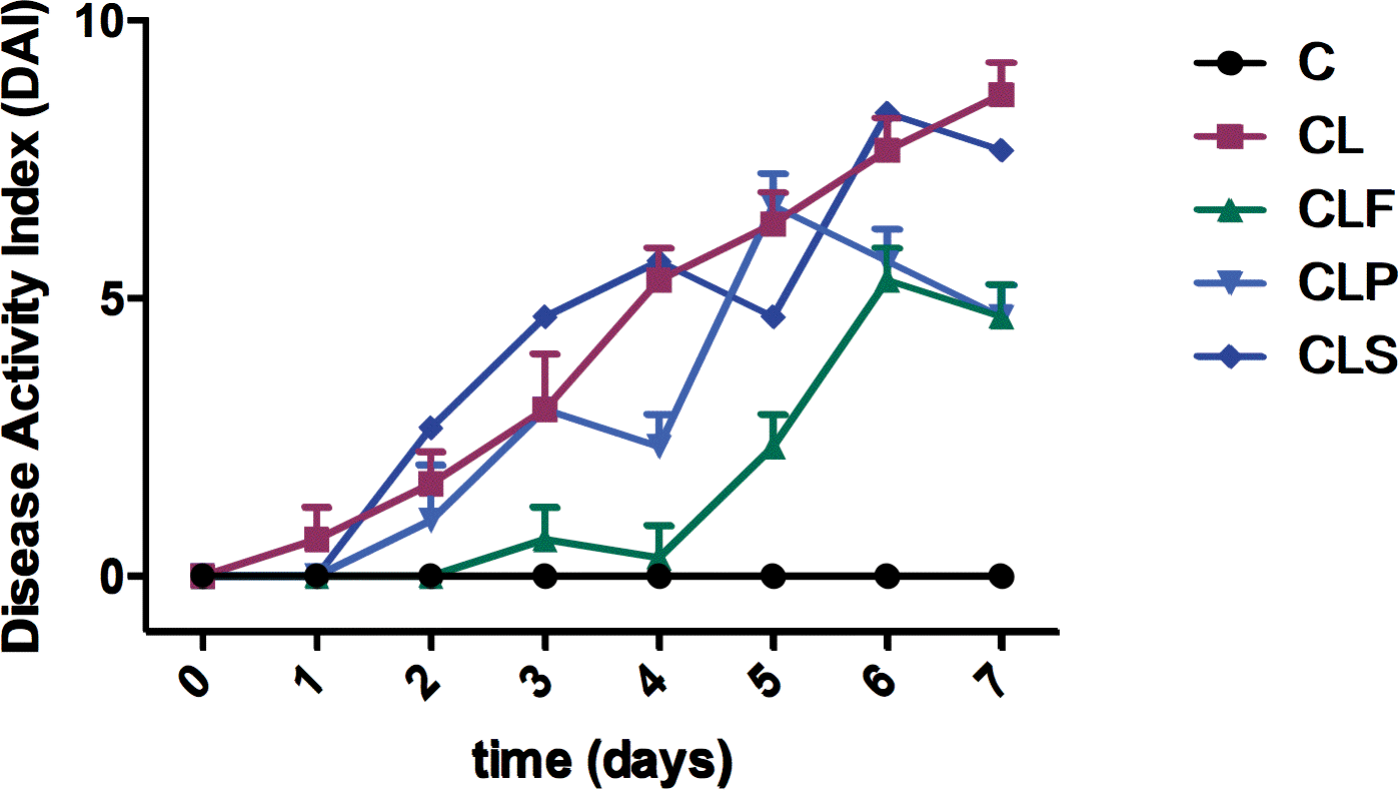
Disease activity index (DAI) of each group during DSS induction period. **Group C:** negative control–healthy animals; **Group CL:** positive control—DSS; **Group CLF:** DSS with the fermented product; **Group CLP:** DSS with the non-fermented product (placebo); **Group CLS:** DSS with sulfasalazine. n = 10.

The behaviors of all the groups were compared to evaluate the development of the disease during the 7 days of induction (T2). There was no statistical difference (p<0.05) among the groups regarding water and food intake (data not shown). The DSS-induced colitis groups (CL, CLP, CLF and CLS) showed signs and symptoms of colitis, as previously indicated, especially by increased DAI during the induction period (Fig 2). These DAI increasements were expected in all induced groups since the DSS acts by irritating the intestinal mucosa causing symptoms similar to ulcerative colitis in patients [53].

The animals from group C showed no weight loss, changes in stool consistency or presence of fecal occult blood during the 7 days of treatment. On the other hand, those belonging to the groups that received water with DSS addition (4%) exhibited weight loss and / or altered stool consistency (loose stool and diarrhea), and / or presence of occult blood in the stool and apparent blood from the third day of induction. These results are consistent with other authors who had used the same method of induction [16,18,54,55].

Among DSS-induced groups, it is noteworthy that the group of rats that consumed the probiotic soy product (CLF) showed fewer changes in the parameters evaluated during the induction period. This was characterized by the lowest weight loss, regular stool consistency until the 4^th^ day and the presence of occult or apparent blood in the stool only on the 6^th^ and 7^th^ days. Even though this group also showed the DSS-colitis symptoms, these results indicate a possible protective effect of the probiotic product being tested herein, since the colitis was clearly less severe under the product administration.

Similarly to our experiment, other recent studies have demonstrated postive effects of different probiotic strains in DSS-colitis. Jo et al. [56] found that mice treated with *Lactobacillus curvatus* WiKim38, a strain isolated from kimchi, had an increased survival rate, fewer clinical signs, and less histopathological severity in comparison with DSS-treated mice not receiving the probiotic.

Moreover, Ahl et al. [57] have demonstrated a reduction in colonic inflammation in C57Bl/6 mice using *Lactobacillus reuteri* R2LC and 5659. This study suggests that these two specific probiotic strains were also able to maintain a thicker, firmly adherent mucus layer compared to the control group. This result demonstrated the ability of these probiotics in strengthening the pre-ephitelial barrier, which works as a physical protection against luminal bacteria.

### CLF and CLP groups display considerable differences in colonic inflammation and crypt pathology

**Fig 3:**
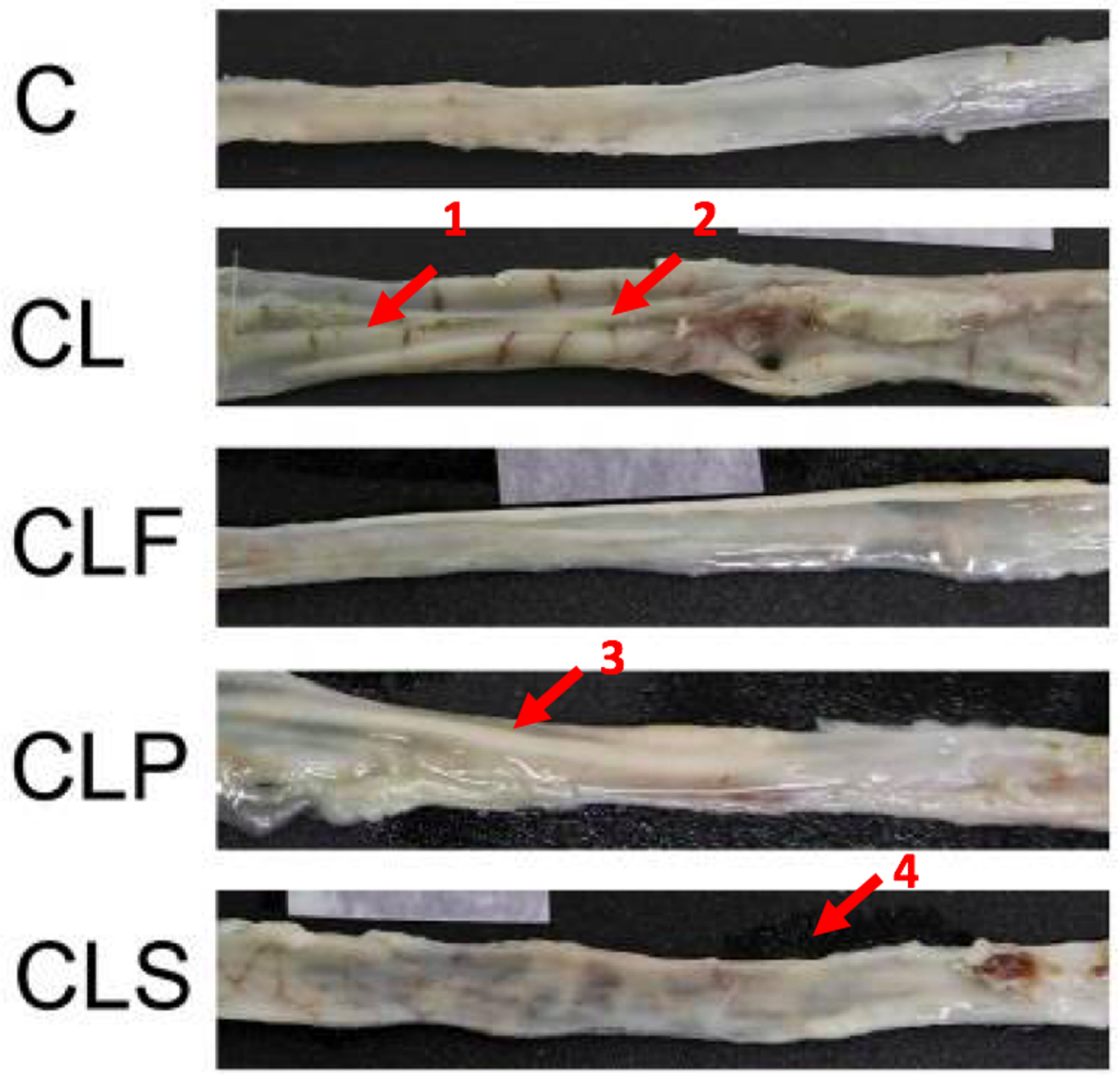
Macroscopic analysis of colon in different groups of animals. **Group C:** negative control–healthy animals; **Group CL:** positive control—DSS; **Group CLF:** DSS with the fermented product; **Group CLP:** DSS with the non-fermented product (placebo); **Group CLS:** DSS with sulfasalazine. **1** and **3:** edema areas; **2** and **4**: ulceration areas. n = 10.

Macroscopic analysis (Fig 3) showed that the colon of animals from CL and CLS groups had swollen and ulcerated areas. In the placebo group (CLP), the swollen areas were observed without the presence of ulcerations. The intestines of animals from the C and CLF groups showed no visible ulcers or swollen areas, thus suggesting that probiotic soy product consumption was able to maintain the integrity of the intestinal mucosa.

The beneficial effect of the probiotic product is evident in the macroscopic tissue according to the scale proposed by Bell et al. [33]. The obtained results (Table 1) indicate that animals that consumed the probiotic product showed a lesser degree of inflammation and / or ulceration when compared to the other colitis-induced groups, thus being significantly different from the CL and CLS groups (p<0.05). The colon weight/length ratio is another parameter that is usually assessed to confirm the severity of colitis, since the damage caused by the disease often results in a swollen and shortened colon. Although significant changes in colon weight/length ratio among the groups were not observed (p>0.05), the CL group showed the highest score in comparison with other groups (p<0.05), indicating a greater tissue damage. Moreover, the CLF group had a very close score to the control group, which might be related to a protective effect to the probiotic soy product.

**Table 1:** Macroscopic analyses of colon damage.

Group	Score	Colon (mg/cm)
**C**	0	127.06±14.62^a^
**CL**	3.44±0.73^a^	142.03±18.85^a^
**CLF**	**0.80±0.63**^**c**^	128.90±15.74^a^
**CLP**	1.20±0.87^bc^	135.93±15.03^a^
**CLS**	2.20±0.97^b^	127.39±9.09^a^

Score of each group was determined according to Bell et al. (1995). The results are presented as mean ± SD. Means with the same letter in the same column do not differ (p<0.05). Statistical analysis for the score was carried out among induced colitis groups. **Group C:** negative control–healthy animals; **Group CL:** positive control—DSS; **Group CLF:** DSS with the fermented product; **Group CLP:** DSS with the non-fermented product (placebo); **Group CLS:** DSS with sulfasalazine. n = 10.

Damage to the colon mucosa is often evaluated as degree of tissue inflammation indicators and crypts hyperplasia may be a compensatory mechanism for the damage in the crypts caused by ulcerations [16,22,58].

**Fig 4:**
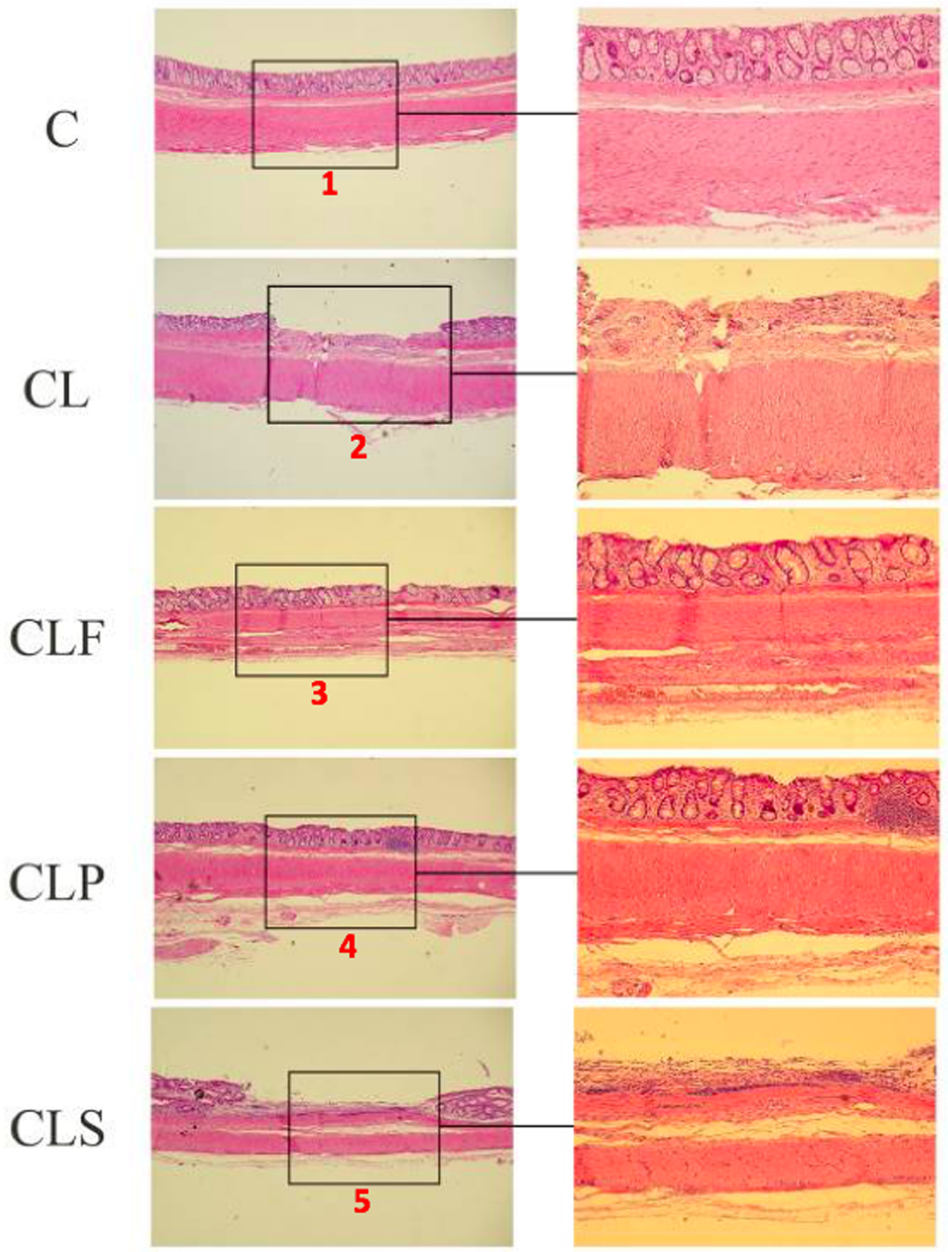
Representative photomicrographs from the different experimental colon groups, stained with hematoxylin/eosin. **Group C:** healthy animals that did not receive the products under study. **Group C:** negative control–healthy animals; **Group CL:** positive control—DSS; **Group CLF:** DSS with the fermented product; **Group CLP:** DSS with the non-fermented product (placebo); **Group CLS:** DSS with sulfasalazine. (100x and 400x). **1:** healthy epithelium; **2 and 5**: epithelium with severe crypt damage, featuring as ulceration area; **3 e 4:** inflammatory cell infiltrated areas, however with no crypt alteration. n = 10.

Fig 4 shows the protective effect of the probiotic product on DSS-induced histopathological changes, since CL and CLS groups presented areas of ulceration, severe crypt damage and epithelial surface erosion which were absent in groups C and CLF.

Colon tissues from the CLF group showed only an infiltration of typical inflammatory cells. Crypt alterations and ulceration areas were not observed in the epithelium, thus suggesting that the probiotic product may have attenuated the severity of the chemically induced inflammation by DSS.

A study conducted by Geier et al. compared the effect of 4 potential probiotic microorganisms in chemically-induced colitis (DSS) in rats, and their results showed that *Lactobacillus fermentum* BR11 was able to alleviate moderately induced colitis in animals. This microorganism reduced the DAI, prevented the shortening of the colon, increased animal weight gains and decreased the risk of crypts hyperplasia [16].

On the other hand, studies were not able to show a positive effect of probiotics on colitis. Zhou et al. [59] verified the effect of *Lactobacillus crispatus* M206119 on the development of DSS-induced colitis in mice. Animals treated with microorganism suspension showed a higher severity of colitis, greater weight loss, diarrhea, bloody stools, decreased colon length, higher histopathological damage and neutrophils infiltration when compared to the control group [59]. This fact indicates that the effect of probiotic microorganisms is strain-specific and that, in rare cases, it may exacerbate disease progressions. Therefore, various future studies are required to verify the efficacy of bacterial strains in animal model before being tested in humans [16].

In the present study, the histopathological analysis also showed that colons in the CLP group had no areas of ulceration and crypt hyperplasia, thus suggesting a protective effect of the soy-based product on colitis development. Positive effects in placebo-treated animals can be partly explained by the soy-based product composition and, consequently, the presence of bioactive components in it, for example isoflavones, which have well-documented anti-inflammatory characteristics [60–64].

In this line of thought, Jiang et al. [65] assessed the effect of a dietary intake of casein, soy protein or whey protein, and *Lactobacillus rhamnosus* GG in DSS-induced colitis in mice. Their results indicate that the soy protein alone can attenuated the degree of inflammation and the expression of TNF, regardless of whether or not the probiotic strain was present. The anti-inflammatory effects observed were contributed by the presence of lunasin, a bioactive peptide present in the soy protein.

Sulfasalazine, the union of 5-aminosalicylic acid (biologically active molecule) and sulfapyridine moiety, is an anti-inflammatory and analgesic non-steroidal drug (NSAID) from the salicylates group, which has been widely used for the treatment of moderate ulcerative colitis [66]. Allergic reactions and various side effects are common in patients taking this type of drug, which occurs in over 33% of patients taking drug maintenance dose and 50% receiving therapeutic doses [67]. Its action mechanism includes reduced production of interleukin1-beta (IL-1β), migration inhibition of polymorphonuclear leukocytes, lipoxygenase cells and production of pro-inflammatory leukotrienes (LTB4 and 5-HETE) by macrophages in the intestinal wall and prostaglandins [68,69]. In this study, sulfasalazine was not able to control the characteristic inflammatory process of colitis, since animals’ colons showed characteristics that are similar to the CL group. A plausible explanation for the obtained result could be due to the short period of treatment (16 days), since drug administration was only conducted after colitis induction.

### Fermented soy product enhances commensal bacteria population

Clinical and experimental studies using different models indicate that it is extremely important to be aware of the intestinal microbiota composition in order to better understand the etiology of ulcerative colitis [70]. Although the pathogenesis of IBD has not been fully elucidated, research indicates that there may be an intolerance to certain commensal bacteria of the intestinal microbiota, which probably cause an unbalance in immune response and results in the development of inflammatory bowel disease [71,72].

Uronis et al. [19] verified the effect of VSL#3 (*L*. *paracasei*, *L*. *plantarum*, *L*. *acidophilus*, *L*. *delbrueckii* subsp. *bulgaricus*, *B*. *longum*, and *B*. *breve*) on the intestinal microbiota of rats with acid-2.4.6-trinitrobenzenesulfonic (TNBS)-induced colitis. The study showed a significant reduction in the severity of chronic colitis in rats that received probiotics when compared to the control group. Furthermore, the results indicated a negative correlation between the severity of colitis inflammation and the diversity of microorganisms in the intestinal microbiota.

Generally at the beginning of the protocol, populations of the studied bacterial group were significantly different (p<0.05). Thus, a comparison among different time points was more important to track possible variation in the microbiota composition during the experiment. Although a statistical data analysis of fecal microbiota composition (p<0.05) has been carried out, changes above 0.5 log CFU/g were considered significant from a microbiological point of view [73].

Table 2 shows the results of *Enterococcus* spp. present in the feces from different experimental groups during the protocol. The control group (C) maintained a bacterial population in the order of 10^5^CFU/g throughout the experiment, while the remaining groups that received the DSS induction varied in magnitude. The population of *Enterococcus* spp. in the CLF group remained stable until the end of the induction period (T2). However, at the end of the protocol (T4) there was a small decrease in this genus in the same experimental group, although this reduction was less than half a log cycle (0.45 log CFU/g).

**Table 2:** Plate counts for different microbial groups expressed in logCFU/g.

	T0	T1	T2	T3	T4
***Enterococcus* spp.**					
**C**	5.67±0.03^a^	5.08±0.04^c^	5.76±0.01^a^	5.30±0.04^b^	5.83±0.16^a^
**CL**	6.34±0.06^a^	4.66±0.08^e^	5.98±0.08^b^	5.18±0.10^d^	5.56±0.04^c^
**CLF**	6.04±0.01^b^	6.06±0.06^b^	6.40±0.11^a^	5.72±0.11^c^	5.59±0.10^c^
**CLP**	5.56±0.04^a^	4.13±0.00^c^	4.80±0.45^b^	5.81±0.04^a^	6.04±0.02^a^
**CLS**	5.90±0.01^ab^	6.03±0.06^a^	5.81±0.09^b^	4.73±0.02^c^	5.85±0.06^b^
***Lactobacillus* spp.**					
**C**	6.83±0.08^a^	6.09±0.09^c^	6.43±0.05^b^	5.78±0.02^d^	6.45±0.02^b^
**CL**	6.65±0.00^a^	6.47±0.05^b^	6.76±0.01^a^	5.95±0.08^c^	6.41±0.01^b^
**CLF**	6.04±0.01^d^	5.86±0.04^e^	6.62±0.01^b^	6.52±0.01^c^	6.88±0.02^a^
**CLP**	6.45±0.05^bc^	5.79±0.05^d^	6.56±0.07^b^	6.42±0.02^c^	6.86±0.04^a^
**CLS**	6.47±0.02^b^	6.03±0.06^c^	6.80±0.08^a^	6.45±0.02^b^	6.81±0.10^a^
***Bifidobacterium* spp.**					
**C**	7.02±0.00^a^	6.58±0.05^b^	6.36±0.10^c^	6.34±0.08c	5.99±0.02^d^
**CL**	6.92±0.01^a^	5.96±0.05^c^	6.52±0.02^b^	5.92±0.01c	5.75±0.09^d^
**CLF**	5.11±0.01^c^	6.00±0.20^b^	6.53±0.08^a^	6.40±0.03a	6.46±0.05^a^
**CLP**	5.90±0.01^c^	5.37±0.07^d^	5.92±0.04^c^	6.39±0.04a	6.17±0.10^b^
**CLS**	6.78±0.01^a^	6.01±0.11^b^	5.39±0.06^d^	5.70±0.02c	5.85±0.06^bc^
***Clostridium* spp.**					
**C**	6.86±0.03^a^	6.41±0.10^b^	6.37±0.16^b^	6.56±0.07^b^	6.10±0.07^c^
**CL**	6.78±0.02^a^	5.72±0.02^d^	6.82±0.04^a^	5.99±0.02^c^	6.41±0.08^b^
**CLF**	6.45±0.08^a^	5.80±0.04^b^	6.48±0.07^a^	6.39±0.06^a^	6.55±0.07^a^
**CLP**	6.40±0.17^b^	5.81±0.06^c^	6.71±0.09^a^	6.48±0.04^ab^	6.65±0.03^a^
**CLS**	6.63±0.04^ab^	6.72±0.03^a^	6.45±0.05^b^	6.52±0.06^b^	6.59±0.11^ab^
**Enterobacteria**					
**C**	3.17±0.06^ab^	2.76±0.03^c^	3.43±0.10^a^	3.05±0.04^bc^	2.91±0.19^bc^
**CL**	3.63±0.11^a^	3.14±0.03^bc^	3.24±0.06b	2.99±0.03^c^	3.27±0.03^b^
**CLF**	3.01±0.00^ba^	2.34±0.31^c^	3.26±0.03^a^	2.85±0.06^b^	2.82±0.08^b^
**CLP**	2.48±0.13^d^	2.65±0.08^cd^	3.27±0.15^a^	2.93±0.04^b^	2.77±0.01^bc^
**CLS**	2.85±0.02^b^	3.06±0.06^a^	2.77±0.14^bc^	2.66±0.00^c^	2.65±0.02^c^
***Bacteroides* spp.**					
**C**	3.02±0.02^b^	3.70±0.01^a^	2.41±0.08^c^	3.02±0.10^b^	3.01±0.09^b^
**CL**	3.03±0.03^b^	3.65±0.06^a^	2.48±0.03^c^	3.08±0.01^b^	3.18±0.02^b^
**CLF**	2.77±0.01^a^	2.53±0.08^a^	2.65±0.08^a^	2.70±0.10^a^	2.59±0.02^a^
**CLP**	2.77±0.07^a^	2.74±0.12^a^	2.46±0.05^b^	2.60±0.02^a^	2.69±0.06^a^
**CLS**	3.77±0.13^a^	2.58±0.13^b^	2.56±0.22^b^	2.48±0.01^b^	2.73±0.02^b^

Means ±SD with the same letter in the same row are not statistically different from each other (p<0.05). **Group C:** negative control–healthy animals; **Group CL:** positive control—DSS; **Group CLF:** DSS with the fermented product; **Group CLP:** DSS with the non-fermented product (placebo); **Group CLS:** DSS with sulfasalazine. **T0** = before products administration, **T1** = one week after products administration, **T2** = colitis induction period, **T3** = one week after the end of the induction **T4** = at the end of the experiment. n = 10.

*Enterococcus* spp. population reduction in the CLF group, despite being low, was unexpected. Nonetheless, the genus *Enterococcus* spp. comprises multiple species of microorganisms, some of them even being considered pathogenic [74], thus the observed alterations do not necessarily mean a decrease in probiotic species.

On the other hand, Bedani et al. [75] has found an increase in the population of *Enterococcus* spp. in mice fed with a soy product fermented with *E*. *faecium* CRL 183 and *L*. *helveticus* 416 or the pure culture of *E*. *faecium* CRL 183. However, the animals fed with the sterilized fermented soybean product also showed an increase in *Enterococcus* spp. genus, thence suggesting that the fermentation process produces certain metabolites that might influence the population of this genus of microorganisms.

Similarly, Cavallini et al. [28] used the same fermented soy product (*E*. *faecium* CRL 183 and *L*. *helveticus* 416) to determine the relationship between rabbits’ fecal microbiota and cardiovascular disease risk factors. After 60 days of experiment, there was a significant increase in the population of *E*. *faecium* in animals that received the fermented soy product, therefore suggesting that this probiotic microorganism might persist in the fecal microbiota during this period.

Some bacterial genera are considered to be beneficial to the host, such as specific strains from the genera *Lactobacillus* spp. and *Bifidobacterium* spp., since they have been identified as important microorganisms with immunomodulatory activities and anticarcinogenic properties [28,75–77].

The results presented in Table 2 indicate that a variation in the population of *Lactobacillus* spp. was less than one log cycle throughout the experimental period for all groups. After the induction period (T2), the CLF, CLP, and CLS groups showed an increase in the *Lactobacillus* spp. population in comparison with the previous period (T1), thus indicating that DSS does not adversely affect the viability of this bacterial genus in the treated groups. Interestingly enough, at the end of the protocol (T4), the group that received the probiotic fermented product (CLF) showed the largest increase in the population of *Lactobacillus* spp. (0.84 log CFU/g) compared to the beginning of the experiment (T0), suggesting a beneficial effect of the product of interest. This variation might be related, at least partially, by the presence of *L*. *helveticus* 416 in the fermented soy product.

A previous study using rabbits as animal models showed that a regular intake of a probiotic soy product fermented with *E*. *faecium* CRL 183 and *L*. *helveticus* 416 (without *B*. *longum* ATCC 15707) also led to an increase (1.27 log CFU/g) of *Lactobacillus* spp. population in the fecal content [28].

Peran et al. [25] reported the effect of *Lactobacillus fermentum* 5716 administration, an isolated strain of breast milk, on the intestinal microbiota of rats with colitis induced by trinitrobenzesulfonic acid. The colitis-induced animals that received the probiotic strain showed a higher population of *Lactobacillus* spp. than the colitis-induced animal group without the treatment. No significant changes were observed in the population of *Bifidobacterium* spp. and potentially pathogenic bacteria such as coliforms and enterobacteria.

Nowadays, the use of probiotics either to reduce or treat bacterial infections is a promising area of research [78]. Bullock et al. [79] found that patients with active ulcerative colitis or in remission had a reduction in the population of *Lactobacillus* spp. In 2009, Wine et al. [80] performed an *in vitro* study to evaluate if *Lactobacillus* spp. strains were able to reduce the invasion of epithelial cells by the microorganism *Campylobacter jejuni*, since this microorganism is the most frequent bacterial cause of enterocolitis in humans. The study indicates that the *L*. *helveticus* R0052 strain was effective in reducing the invasion of epithelial cells by two strains of *C*. *jejuni*. Although this study can not be compared to *in vivo* models these findings are relevant to an initial understanding of the interaction between an enteric pathogen and potentially beneficial bacteria.

Studies indicate that the increase of *Bifidobacterium* spp. population in the colon usually brings benefits to their host, such as immune system modulation, production of SCFA and peptides production, and intestinal pH decrease. Furthermore, this genus is also related to the inhibition of growth and adhesion of pathogenic *E*. *coli* to enterocytes [81]. Moreover, Mylonaki et al. [82] reported that a reduction in the population of *Bifidobacterium* spp. is associated with the colorectal epithelium of patients with ulcerative colitis compared to control group patients.

According to Table 2, groups C, CL, and CLS have had a significant reduction of about one log cycle in the population of *Bifidobacterium* spp. throughout the experimental protocol, while the CLP group showed no significant change over the same period. Nevertheless, the group that consumed the probiotic product (CLF) showed a significant increase in the population of *Bifidobacterium* spp. over than one log cycle (1.35 logCFU/g), probably due to the addition of *B*. *longum* ATCC 15707 to the product.

Bedani et al. [75] and Cavallini et al. [28] also reported an increase of *Bifidobacterium* spp. population in animals that consumed the same fermented soy product without the addition of *B*. *longum*, which suggests that these probiotic microorganisms beneficially modulate the fecal microbiota.

In 2011, Messaoudi et al. [83] investigated the possible effects of probiotics consumption by animals and humans suffering from anxiety, depression and stress, since previous studies had related these symptoms to cases of IBD. It has been found that the use of a probiotic formulation (*L*. *helveticus* R0052 and *B*. *longum* R0175) combined with psychological treatment were able to reduce the symptoms and indirectly diminish the incidence of IBD. This can be attributed to a decrease in pathogenic bacteria in the gut microbiota, thus downregulating pro-inflammatory cytokines and inactivating the transmission in the central nervous system via vagal sensory fibers and neurotransmitters [84–86].

Some studies have indicated that the decrease of *Clostridium* spp. population can bring benefits to the organism, since the metabolic activity or the pathogenic characteristics of some species may be harmful to the host’s health [75,87]. In addition, the *Clostridium* spp. and *Bacteroides* spp. are commonly related to inflammatory bowel disease [75,88].

In this study, there was a little variation in the population of *Clostridium* spp. in all groups at the end of the experiment (< 0.5 log cycle), and the probiotic soy product did not affect the clostridia population (Table 2). These results are similar to those obtained by the present research group in a study using rabbits as animal model [28].

Another group of bacteria investigated in the feces of the animals was enterobacteria (Table 2), which comprised several potentially pathogenic bacteria such as *Escherichia coli*, *Salmonella* spp., *Shigella* spp., *Yersinia enterocolitica*, *Klebsiella* spp., *Proteus* spp., and *Citrobacter* spp. [75]. A study conducted by Sokol et al. [89] found a higher prevalence of *E*. *coli* in the feces of patients with ulcerative colitis if compared to healthy patients (control group).

Other studies have shown that the consumption of *Lactobacillus plantarum* decreased the population of enterobacteria in animals, including species of the genera *Escherichia* spp. and *Salmonella* spp., reduced bacterial translocation across the intestinal epithelium and modulated the immune system, thus controlling anti and- pro-inflammatory responses. In humans, *L*. *plantarum* was able to reduce the severiry of irritable bowel syndrome, including abdominal cramps and bloating [90,91].

A previous study has noted a reduction of over 3 logarithmic cycles with the administration of the same probiotic product, without the addition of *B*. *longum*, in an animal model using rabbits [28]. However, a similar effect could not be verified in this study. Despite that variations in the *Enterobacteriaceae* population were significant (p<0.05) at the end of the protocol (Table 2), they are not relevant from a microbiological point of view (<0.5 log CFU/g). This result can be explained by the fact that the animal model used in the study is free of specific pathogens (SPF), thus not showing pathogenic bacteria which are normally found in animals belonging to the same species.

The population of *Bacteroides* spp. remained low (2.65 to 3.08 log CFU/g) and there was no significant changes throughout the experiment, except for the group receiving sulfasalazine, which showed a reduction of approximately one log cycle at the end of the study (Table 2).

Some studies associate *Bacteroides* spp. with an increased risk factor for colon cancer, since certain metabolites produced by these microorganisms can be harmful to the host. Additionally, some species such as *Bacteroides fragilis* are related to the etiology of diarrhea in animals and humans [44,92,93].

Recently, several studies have demonstrated that the gut microbiota plays an key role in IBD [94–96]. It seems that gut microbiota diversity is seriously affected in IBD patients and mucosal bacterial numbers are generally increased due to colonic inflammation [94].

It is well known that commensal bacteria are able to prevent infection by pathogens [97]. Despite an undefined mechanism of the protective effect of the commensal bacteria, the use of certain probiotics has been proposed as a strategy to suppress intestinal burden or colonization of pathogenic bacteria [81]. Considering the increase in *Lactobacillus* spp. and *Bifidobacterium* spp. population, the present results are in agreement with these hypotheses and may explain the reason why the CLF group has shown less symptoms and histological alterations compared to the other groups under investigation.

### Fermented soy product modulates cytokine levels and NO expression in DSS-colitis

**Fig 5:**
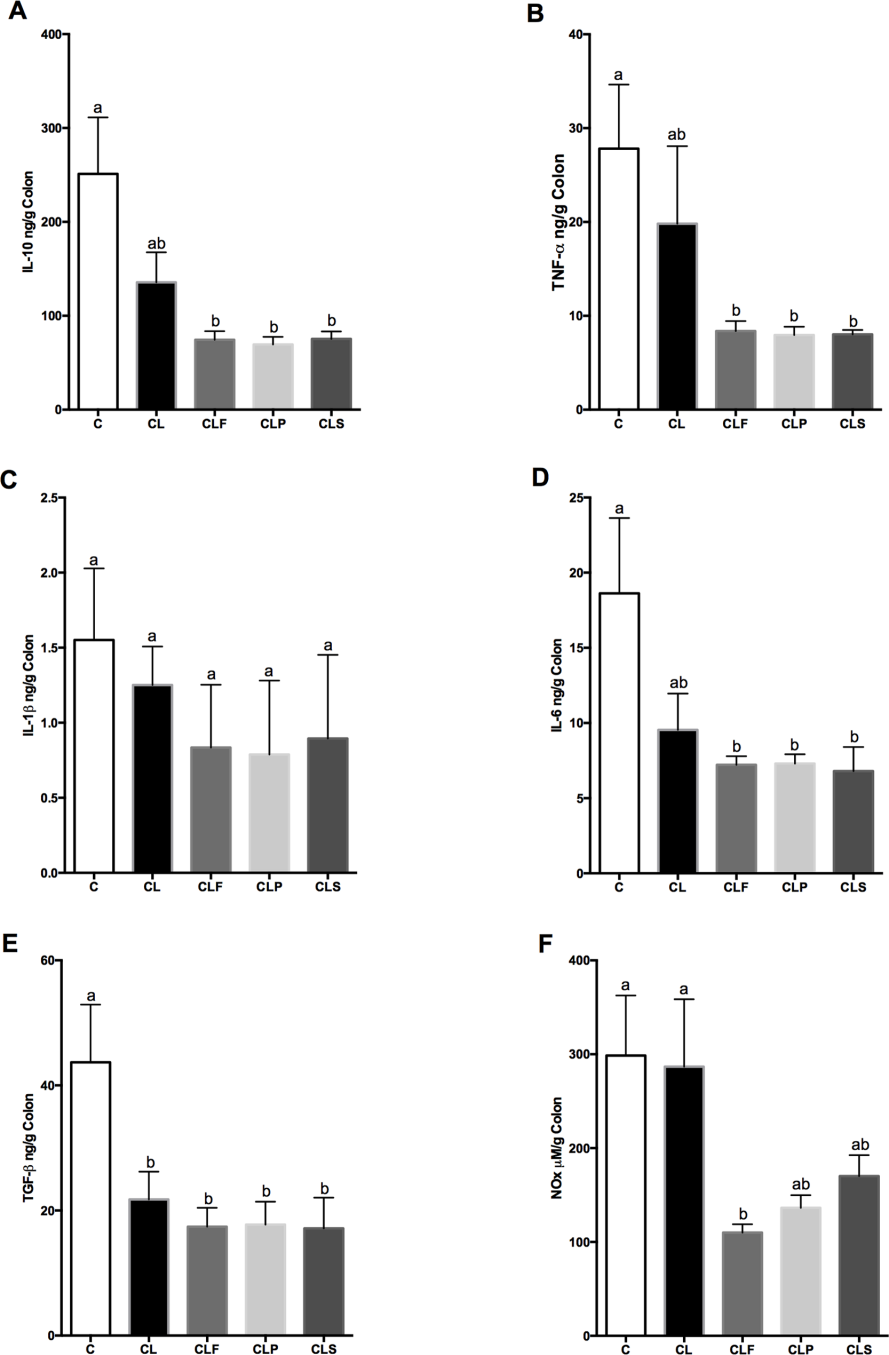
Cytokines production in colon from different groups after 30 days of experimental protocol. **Cytokine in colon.** Concentrations of TNF-α (A); IL-1β (B); IL-6 (C); TGF-β (D); IL-10 (E) and NO (F) were measured by ELISA in colon supernatants. Data are presented as mean ± SD. Means with the same letter in the same graphic do not differ (p<0.05) **Group C:** negative control–healthy animals; **Group CL:** positive control—DSS; **Group CLF:** DSS with the fermented product; **Group CLP:** DSS with the non-fermented product (placebo); **Group CLS:** DSS with sulfasalazine. n = 10.

It was evaluated whether the probiotic fermented soy product was able to modulate anti- and pro-inflammatory cytokines involved in IBD pathophysiology. There were no significant differences in IL-10, TNF-α, IL-1β, IL-6 and NO levels between the control (C) and DSS-colitis group (CL) (Fig 5A–5D and 5F). However, levels of TGF-β were reduced in the DSS-induced colitis group (CL) compared to the control group (C) (Fig 5E). These results were unexpected since studies have suggested that pro- and anti-inflammatory cytokines imbalance is directly related to IBD pathophysiology [98].

In this context, the factors that could influence the cytokine profile and explain the results obtained in the present study for control (C) and DSS-induced colitis (CL) groups include animal model and the disease phase evaluated [99–102]. A study conducted by Bento et al. [99] demonstrated that the most relevant differences in cytokines expression occur during the acute phase of DSS-colitis. On the other hand, during the chronic or remission phase, inflammatory cytokines such as TNF-α, IL-6 and IL-1β, decrease drastically to levels almost similar to those found in healthy mice [99]. It is noteworthy that we have evaluated all cytokines expression in colon tissue during the late phase of DSS-colitis (14 days after DSS suspension), which could explain the reason why statistic differences regarding cytokines levels between the C and CL groups were not able to be verified.

TNF-α is an important inflammatory mediator in ulcerative colitis (UC). However, this cytokine is mainly present in the acute phase of the inflammatory process, since it was evaluated the remission colitis phase [99]. It is certain that IL-1β is considered a potent inflammatory mediator and is usually increased in IBD patients [99,103]. However, it was not observed any differences in the levels of IL-1β among the control and colitis-induced groups during the remission phase of the disease in this study.

In addition, the levels of IL-10, TNF-α, IL-6, and TGF-β were significantly reduced in DSS-induced colitis animals that received any type of treatment (CLF, CLP, CLS) compared to control animals (C), whereas no differences were observed when compared to untreated DSS-induced colitis group (CL) (Fig 5A, 5B, 5D and 5E). It is important to consider that the probiotic-treated group (CLF) showed a significant reduction of NO levels compared to untreated DSS- induced colitis (CL) and control groups (C) (Fig 5F).

Experimental IBD animal models have demonstrated that constitutive and inducible NO synthesis during acute colitis can be beneficial, whereas sustained NO overproduction promotes intestinal damage [104]. Moreover, the inflammation in the colon in animal models or in human IBD is related to continuous upregulation of NO synthesis. Indeed, it was demonstrated that treatment with NOS inhibitors in experiment colitis undermines the intestinal inflammation in rodents by a variety of different agents [105–107]. It was also observed that the probiotic treatment reduced NO levels in DSS-induced colitis animals and this may be one of the mechanisms involved in the decreased risk of developing inflammation in such animals.

Several researches using *in vivo* models of IBD have showed that a daily intake of probiotic strains result in an increased expression of the anti-inflammatory cytokine IL-10 [24–27]. Although higher IL-10 levels were not observed in probiotic-treated groups, the result suggest that an ingestion of the probiotic and the non-fermented products may down regulate different cytokine production. In addition, TNF-α was decreased in probiotic-treated groups and histopathological studies also indicate a better restoration of the colonic tissue in these animals and leading to a quicker recovery of the colon. Nevertheless, further studies are necessary to certify and explain the results regarding the fermented soy product effect on pro- and anti-inflammatory cytokines modulation.

### Upregulation of fecal short chain fatty acids

**Fig 6:**
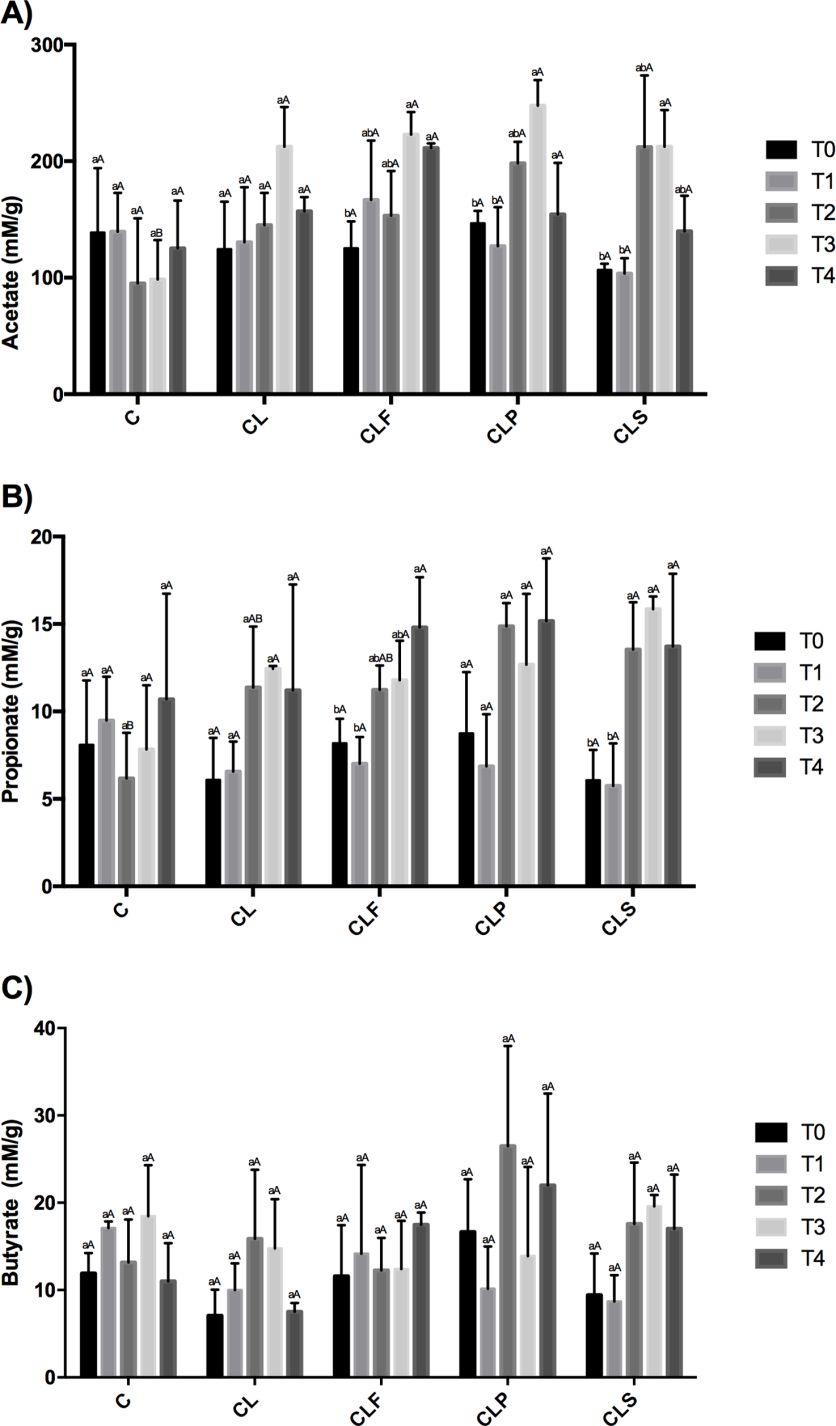
Fecal SCFA concentration (mM/g) from different groups throughout the experimental period. Same lowercase letters indicate that there was no difference when comparing times. Same capital letters indicate that there was no difference when comparing groups (Tukey test, p<0.05). **Group C:** healthy animals that did not receive the products under study. **Group C:** negative control–healthy animals; **Group CL:** positive control—DSS; **Group CLF:** DSS with the fermented product; **Group CLP:** DSS with the non-fermented product (placebo); **Group CLS:** DSS with sulfasalazine. **T0** = before products administration, **T1** = one week after products administration, **T2** = colitis induction period, **T3** = one week after the end of the induction **T4** = at the end of the experiment. **A** = acetate, **B** = propionate, **C** = butyrate.

Acetate, propionate, and butyrate are SCFAs present in large quantities in the colon and result from the bacterial fermentation of carbohydrates that are typical in this intestinal area [108]. The amount of SCFA can be essentially influenced by three factors: diet composition, intestinal microbiota and intestinal area [108,109]. SCFA are not only considered an important substrate for the enterocytes, but are also able to modulate the inflammatory response [25,110,111]. Fig 6 shows SCFA concentration in the animals’ colon.

C and CL groups showed no significant difference (p<0.05) throughout the experimental protocol in all SCFA investigated. This result indicates that the DSS did not affect the production of acetate, propionate and butyrate.

Animals in the CLF group exhibited increased concentrations of acetate and propionate during the trial period, especially just after the induction of colitis. The regular intake of the placebo product (CLP) resulted in an increase in the acetate concentration (p<0.05) only for T3 (one week after the colitis induction). There was no significant change in propionate concentration in the CLP group during the protocol. CLS group presented a significant increase (p<0.05) in acetate and propionate concentration after the induction period, and this effect persisted for propionate until the end of the experiment. There was no statistical difference (p<0.05) in butyrate production throughout the trial period for all groups.

By comparing all groups, the DSS-induced colitis animals (either treated or not), showed higher levels of acetate compared to the control group within a week after the induction (T3). As regards propionate, animals belonging to the CLP group exhibited an increased product yield of this SCFA after the induction period (T2), which differed in the control group only.

Nowadays, studies have suggested that an increase in SCFA, particularly butyrate, plays a positive role in immune system modulation, therefore in inflammatory diseases treatment. About 80% of butyrate is metabolized by enterocytes, thence it is the primary substrate of intestinal cells. However, much of butyrate is extracted by the liver, leaving its plasma concentration at relatively low levels [108,112,113]. Acetate, although being the most abundant SCFA, is rapidly absorbed and transported by the liver, where it will be useful in different metabolic pathways, whereas propionate remains at low concentration in the blood stream after passing through the liver [112,113].

Segain et al. [114] evaluated the effect of butyrate obtained on intestinal cells biopsies of patients with Crohn's disease and found a reduction in the expression of pro-inflammatory cytokines by inhibiting nuclear factor kappa B (NFκB). Peran et al. [25] tested *Lactobacillus fermentum* species in TNBS-colitis rats. They observed an increase in SCFA concentration and increased regeneration of the intestinal mucosa in the group that consumed the probiotic product, thus indicating a positive relationship between this microorganism and SCFA production. In 2008, Nassri et al. [115] verified the effect of a SCFA solution irrigation on mice with exclusion colitis, disease described as an inflammation in colon segments which are devoid of fecal stream. The results showed a decrease in the inflammatory process in addition to a smaller vascular congestion and collagen deposition in the group that received the SCFA solution. The positive effect demonstrated in their study indicates that SCFA are capable to assist in the healing of colon inflammation since they act as important energy substrates for colonocytes [115,116].

Despite the positive effects observed, the fermented product (CLF) was not able to promote a significant increase in fecal butyrate during the study period. This result can be partially attributed to the fact that this acid concentration is unstable in the colon. The majority amount of butyrate is used immediately by colonocytes as a source of energy, for restoring mucosal integrity, or even by their own bacteria in the intestinal microbiota [117].

A recent study conducted by Morampudi et al. [118] discussed a similar result founded by the present research group. According to their findings, Mucin2 deficient mice showed significant increased acetate and propionate levels, but not butyrate, when treated with *Lactobacillus* spp. strains. Apparently, this increase in SCFA can be associated with mucosal healing in Muc2 deficient mice, and perhaps that helps explaining the reason why the CLF group was the most promising approach in the current experiment.

### Gastrointestinal resistance of *E*. *faecium* and *B*. *longum*

To exert its beneficial effect in the body, a probiotic microorganism must be able to adhere to the epithelial mucosa and colonize the gut at least temporarily [119–120]. For better understanding the microbiological results, a molecular analysis was carried out to identify which species of *Enterococcus* spp. and *Bifidobacterium* spp. genus were found in the animals’ feces. Colonies of *Enterococcus* spp. and *Bifidobacterium* spp. with different morphologies were selected and evaluated by using biochemical tests with API 20-Strep and API 50 CHL, respectively.

**Fig 7:**
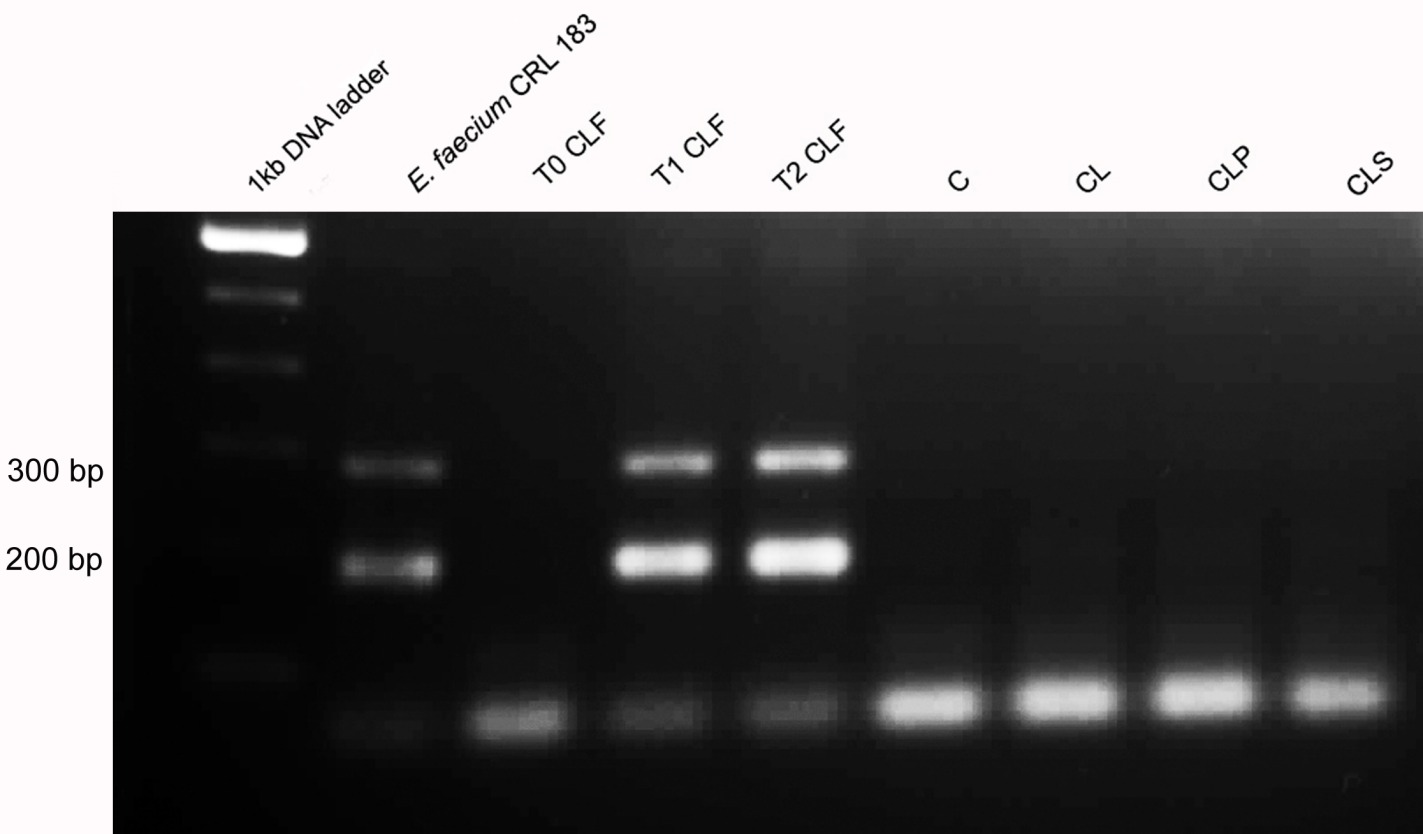
Agarose gel electrophoresis of PCR products obtained from colonies isolated from feces in different groups of animals. Column 1: 1kb DNA ladder; column 2: *E*. *faecium* CRL 183; column 3: T0 CLF group (before products administration), column 4: T1 CLF group (after 2 weeks of products administration); column 5: T2 CLF group (after 4 weeks of products administration); column 6: group C; column 7: group CL; column 8: group CLP; column 9: group CLS. **Group C:** negative control–healthy animals; **Group CL:** positive control—DSS; **Group CLF:** DSS with the fermented product; **Group CLP:** DSS with the non-fermented product (placebo); **Group CLS:** DSS with sulfasalazine.

At the beginning of the protocol, the species of *Enterococcus* spp. identified in the CLF group were *E*. *faecium*, *E*. *faecalis* and *E*. *avium*. This result showed that the species found were already part of the animals’ intestinal microbiota, since no product had been administered yet. The same species were confirmed in the samples collected in the middle of the experiment, which indicates that the DSS did not influence the diversity of *Enterococcus* species. At the end of the experiment, the predominant colonies were *E*. *faecium*, thus suggesting that the strain administered to the fermented soy product was able to survive to the conditions of the gastrointestinal tract and possibly colonized temporarily the gut of animals belonging to the CLF group. This hypothesis was confirmed by molecular biology (PCR) using the specific primer for *E*. *faecium* and using the CRL 183 strain as control. After product administration, only the colonies from the CLF group were confirmed by PCR as belonging to the strain *E*. *faecium* CRL 183. Samples of other groups were not confirmed as belonging to the CRL 183 strain (Fig 7).

The colonies isolated from other groups (C, CL, CLP and CLS) were identified as belonging to the *S*. *uberis*, *E*. *faecalis*, *E*. *faecium*, and *E*. *avium* species within all analyzed time points. It should be noted that it was not observed a diversity shift of species identified during the trial period.

As regards *Bifidobacterium* spp. it was tested its ability to ferment different types of carbohydrates through the API 50 CHL system. Although the API 50 CHL system is not specific for this bacterial genus the identification of species was based on the carbohydrate fermentation profile of each *Bifidobacterium* spp. species (literature data) [121–122]. In the present study, the fermentation profile of a pure strain *B*. *longum* ATCC 15707 was determined for comparison purposes.

**Fig 8:**
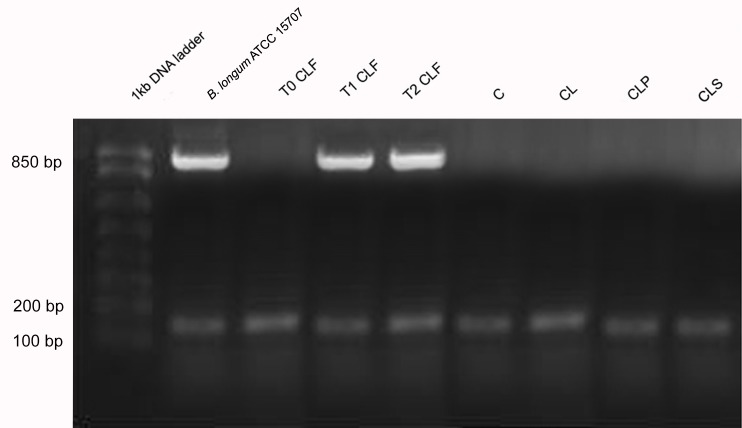
Agarose gel electrophoresis of PCR products obtained from colonies isolated from feces in different groups of animals. Column 1: 1kb DNA ladder; column 2: *B*. *longum* ATCC 15707; column 3: T0 CLF group (before products administration), column 4: T1 CLF group (after 2 weeks of products administration); column 5: T2 CLF group (after 4 weeks of products administration); column 6: group C; column 7: group CL; column 8: group CLP; column 9: group CLS. **Group C:** negative control–healthy animals; **Group CL:** positive control—DSS; **Group CLF:** DSS with the fermented product; **Group CLP:** DSS with the non-fermented product (placebo); **Group CLS:** DSS with sulfasalazine.

All strains of *Bifidobacterium* spp. genus in the CLF group demonstrated, at the beginning of the experiment, the ability to ferment carbohydrates that are typical of the *B*. *longum*, *B*. *bifidum* and *B*. *infantis* species. At the end of the protocol carbohydrates used in fermentation were characteristic of *B*. *longum* species. The isolated colonies of the other groups (C, CL, CLP and CLS) were identified as belonging to the species *B*. *bifidum*, *B*. *infantis*, *B*. *longum*, *B*. *animalis*, *B*. *breve* and *B*. *adolescentis* and there was no change in the diversity of species identified during the trial period. The results suggest that the strain *B*. *longum* ATCC 15707 administered in the soy-based fermented beverage was able to survive the harsh conditions of the gastrointestinal tract of animals belonging to the CLF group (Fig 8).

In general this study showed that the consumption of the probiotic fermented product resulted in an increased population of *Lactobacillus* spp. and *Bifidobacterium* spp. and higher propionate and acetate fecal levels, which is considered important to maintain the integrity of the intestinal epithelial cells. This effect is in agreement with literature data that evidences that *Bifidobacterium* spp. is able to produce acetate and propionate and that these SCFAs can act as a substrate for producing butyrate in the colon [123,124]. The microbiota modulation and production of SCFA are related to the immune response improvement. However, in the present study there was no clear relationship between the composition of the microbiota and the concentration of pro- and anti-inflammatory cytokines probably due to the phase of the disease evaluated. Thus, future studies on different stages of colitis and using different IBD models should be conducted to elucidate the mechanisms and confirm the beneficial effects of the probiotic fermented product.

## Conclusion

The results of this study indicate that a regular intake of the fermented probiotic soy product (CLF) could be considered as an option to reduce the risk of DSS-colitis in an animal model. Although future studies are needed to confirm this beneficial effect, this finding may be a promising approach for IBD patients since the product has no adverse effects and could be easily incorporated into their regular daily diet.
